# Design of Multivalent Inhibitors for Preventing Cellular Uptake

**DOI:** 10.1038/s41598-017-11735-7

**Published:** 2017-09-15

**Authors:** Veronika Schubertová, Francisco J. Martinez-Veracoechea, Robert Vácha

**Affiliations:** 1 Faculty of Science, Masaryk University, Brno, Czech Republic; 2 Central European Institute of Technology, Masaryk University, Brno, Czech Republic; 3 Eesti Energia AS, Lelle 22, 11318 Tallinn, Estonia

## Abstract

Cellular entry, the first crucial step of viral infection, can be inhibited by molecules adsorbed on the virus surface. However, apart from using stronger affinity, little is known about the properties of such inhibitors that could increase their effectiveness. Our simulations showed that multivalent inhibitors can be designed to be much more efficient than their monovalent counterparts. For example, for our particular simulation model, a single multivalent inhibitor spanning 5 to 6 binding sites is enough to prevent the uptake compared to the required 1/3 of all the receptor binding sites needed to be blocked by monovalent inhibitors. Interestingly, multivalent inhibitors are more efficient in inhibiting the uptake not only due to their increased affinity but mainly due to the co-localization of the inhibited receptor binding sites at the virion’s surface. Furthermore, we show that Janus-like inhibitors do not induce virus aggregation. Our findings may be generalized to other uptake processes including bacteria and drug-delivery.

## Introduction

Nano-inhibitors that can selectively target viruses and prevent them from infecting cells could be a game changer in the development of antiviral therapeutics and could have a huge impact in the treatment of challenging diseases caused by viruses like Dengue, Influenza, Ebola, and Zika^1,2^. To infect a target cell, a virus typically needs to bind to receptors on the cellular membrane triggering the internalization via receptor-mediated endocytosis. Hence, one way to prevent viral infection is by developing inhibitors that can effectively and selectively bind to the virus capsid before they can bind to the cell membrane receptors and stop the internalization.

During internalization, the virus is wrapped by the membrane. In this process, the bending energy needs to be compensated by the interaction with receptors and can be stabilized by protein assemblies^3–14^. This is a very general mechanism that can be either active (i.e., where the expenditure of ATP is necessary) or passive (i.e., no ATP needed). The active process is the most recognized for viruses, yet uptake determined by lipids as receptors without signaling was also reported^15,16^. The internalized objects can have various shapes (e.g., spheres, icosahedrons, or elongated particles) that affect the specific uptake path^3,11,17,18^. However, all shapes require having receptor binding sites (RBS) (e.g., ligands or binding pockets) on their surface in order to be uptaken, and the spatial distribution of such sites can be critical for determining the internalization efficiency^6,12^.

**Figure 1:**
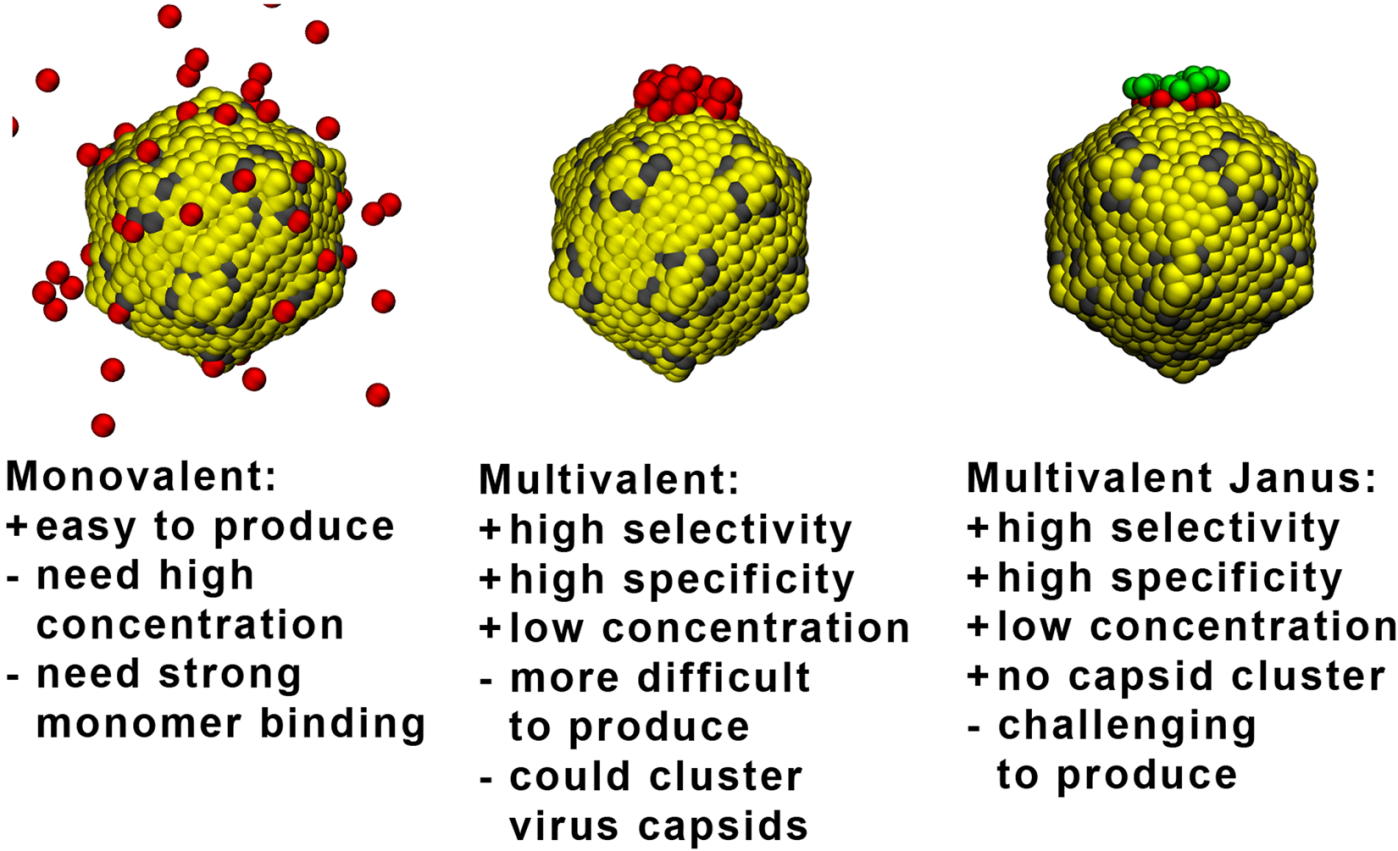
Representative snapshots of a model virus capsid in the presence of different inhibitors. Advantages and disadvantages of each inhibitor type is described.

It is well known that multivalent entities (e.g., polymers, star-polymers, nanoparticles, etc.) can be designed to bind selectively and with strong affinity to RBSs on a surface^19–21^. Therefore, it is only natural to exploit multivalency in the development of viral-inhibitors^22–24^. In the present study we show that multivalent inhibitors can provide additional advantages besides super-selectivity and increased affinity: they can be designed for spatially correlated targeting of RBSs on the virus capsid. We show that spontaneous endocytosis of a virus is most efficiently hindered when the blocked RBSs are spatially close to each other. While creating such an “inhibited patch” can prove very difficult with monovalent inhibitors, this task can be readily achieved by multivalent inhibitors. Moreover, we demonstrate that Janus multivalent inhibitors (with inhibitors on one side and inert on the other side) could also be the best option for preventing inhibitor-bridged aggregation and uptake of capsids (see Fig. 1).

## Results and Discussion

**Figure 2:**
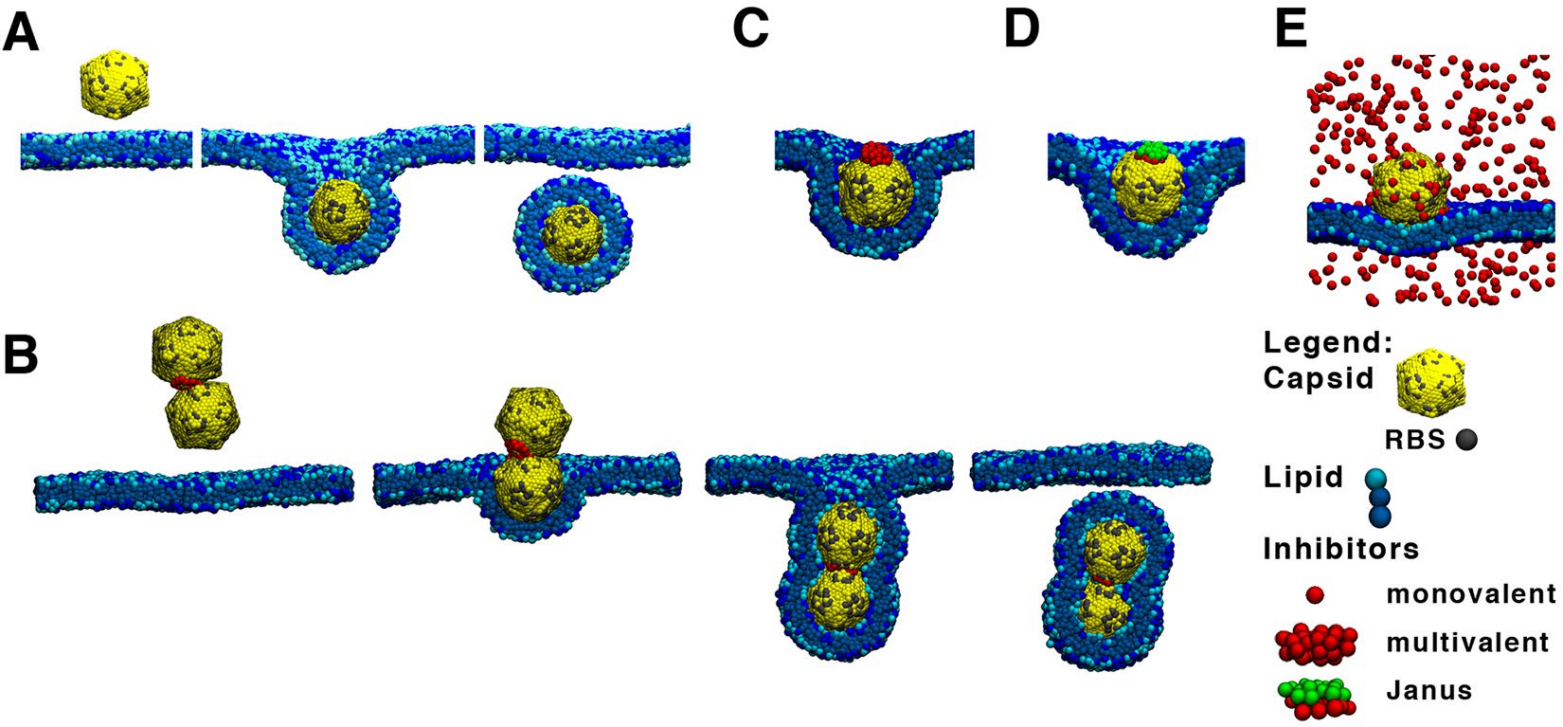
(**A**) Representative snapshots from uptake trajectories of model virus capsids. The stages are binding, partial encapsulation, and full uptake. (**B**) Two capsids are bound together by multivalent inhibitor and are uptaken together. Stopped uptake by (**C**) multivalent or (**D**) Janus multivalent inhibitors (**E**) many monovalent inhibitors. A cut through the membrane at capsids positions is depicted for clarity.

In Fig. 2 we show the typical behaviour of monovalent and multivalent inhibitors. Inhibition by monovalent entities required a large number of the virus’s RBSs to be bound by inhibitors (i.e., a large fraction of the RBSs needed to be blocked) to prevent the uptake. To achieve such high RBS coverage, it is necessary to have either very high bulk concentration of inhibitors in solution or extremely strong inhibitor-receptor interaction (for details see Supplementary Information).

In stark contrast, a single copy of our model of the multivalent inhibitor is required﻿ to frustrate endocytosis in our simulations. Interestingly, the multivalent inhibitor stops the uptake when it is almost fully wrapped by the membrane (Fig. 2E). In the case of monovalent inhibitors, the degree of wrapping depends on the virus’s surface degree of coverage by the inhibitors and at high coverage it can even stop the uptake with only little membrane bending (see Supplementary Information). This can be explained based on the geometry and energetics during the wrapping process^12^. As the insertion depth increases the number of RBSs that need to be in contact with receptors also increases to counter the energetic penalty due to the membrane’s bending. In the monovalent inhibitor case, the viruses RBSs are inhibited more or less uniformly across the virus’s surface. Therefore, uptake is hindered when the attraction between virus and membrane can no longer compensate the penalty for membrane bending. When multivalent inhibitors are used, inhibition occurs heterogeneously on the virus surface. Wrapping is only halted when the virus is almost fully covered by the membrane at which point the membrane reaches the inhibitor. At this stage, extra-bending is no longer rewarded by virus membrane contacts and the uptake process is halted.

It has been previously observed^12^ that the distribution of active RBSs (i.e., those that can directly bind to the receptors on a cellular membrane) can have a profound effect on the endocytosis process. More specifically, for two nano-objects with the same number of RBSs, the one with the most homogeneous distribution of RBSs can be uptaken more easily. Moreover, nano-objects with large-enough RBS-free patches were not uptaken. Hence, we are led to expect that the difference in efficiency between monovalent and multivalent inhibitors mainly stems from the fact that monovalent inhibition is spatially uncorrelated while multivalent agents can be designed to inhibit RBSs in localized areas (i.e., patches). This is because while monovalent inhibitors act independently of each other, in the multivalent case the inhibition moieties are constrained (by the architecture of the multivalent inhibitor) to remain localized. In addition, it is expected that the increase in affinity due to multivalency will greatly reduce the concentration needed in solution for the inhibitor to attach to the virus.

In order to provide a quantitative study of the difference in efficiency between monovalent and multivalent inhibitors, ideally, we would perform a series of simulations where we explicitly model the inhibitors and observe the dynamics of virus uptake for sufficiently long (simulation) time. In practice, however, there are problems with this approach (see SI): (a) the number of monovalent inhibitors in solution needed to achieve good virus coverage and not affecting the bulk concentration is very large, making the simulations slower (b) As the simulation progresses and the monovalent inhibitors attach to the virus the bulk concentration of inhibitors does not remain constant making results difficult to interpret. (c) The results depend on features of the inhibitor model such as inhibitor size, binding strength, etc. that are out of the scope of the current work. (d) For the multivalent case, it is hard to decouple the classical multivalent effects (i.e., selectivity and increased affinity) from the co-localization effect.

To address all these problems, we devised simulations where we mimicked the effect of the inhibitors without explicitly simulating them. In these simulations, we simply inhibited/deactivated selected RBSs on the virus surface and observed how the uptake process was affected. In the case of mimicking monovalent inhibition the RBSs were deactivated randomly (i.e., without considering its position). In the case of mimicking multivalent inhibition, the deactivation was carried over a localized patch (see Appendix and SI for details). With this approach, we can make quantitative predictions within reasonable simulation time. Moreover, since the relationship between inhibitor concentration and the number of inhibited RBSs depends on the inhibitor model details (e.g., binding strength, valence, architecture, etc.) we are making our results more general by focusing primarily on the number of inhibited sites. One disadvantage of this approach is that by making our inhibitors implicit we neglect the effect of steric repulsion on the inhibition process. However, as mentioned before, steric effects are largely model dependent (e.g., size, architecture, etc.) and are outside of the scope of the current work.

**Figure 3:**
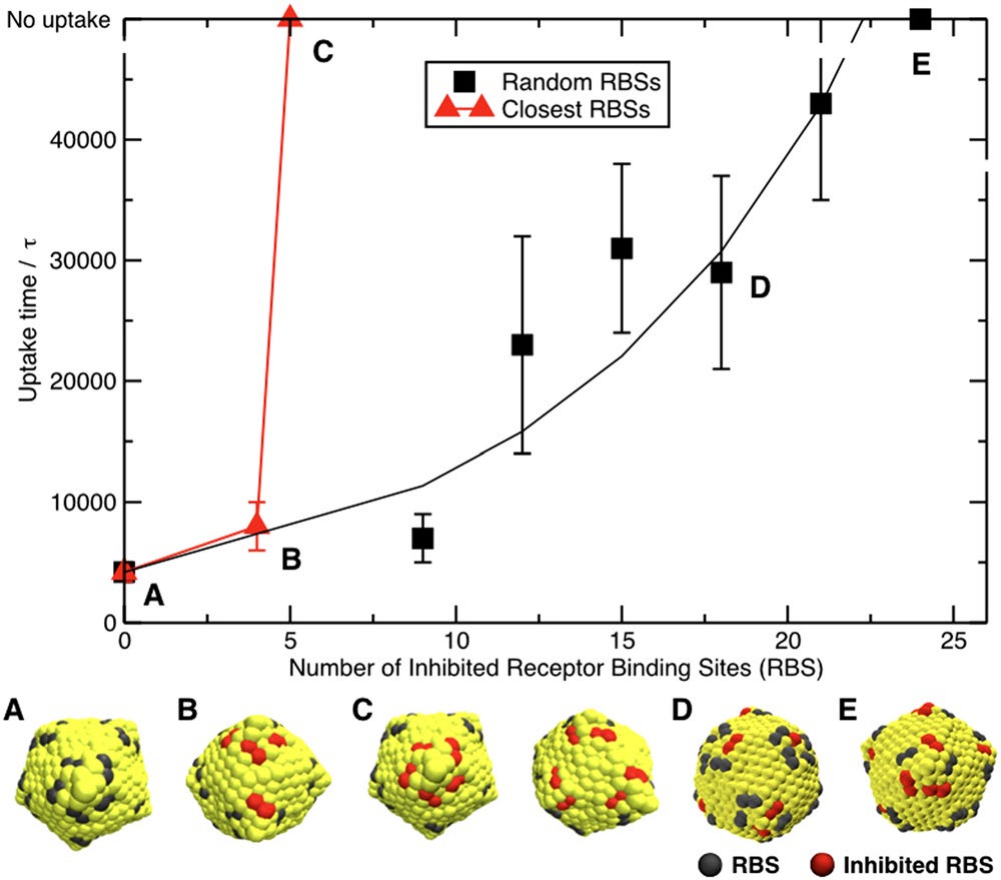
Dependence of uptake time on the number of inhibited sites distributed randomly (black squares) or closest to each other (red triangles) on the virus capsid. These situations correspond to monovalent and multivalent inhibitors respectively. Black line is an exponential fit to guide the eye, the red line is only the connection between the points due to the small number of points. Illustrative snapshots of virus like capsid with 60 RBSs and defined number of inhibited RBSs are depicted for specific points. Capsid color coding: yellow - hydrophilic beads, gray - active RBSs, and red - inhibited RBSs.

The uptake times of both, randomly-located and localized, inhibited sites are depicted in Fig. 3. The fully active capsid with all 60 bindings sites available (RBS60) was wrapped and uptaken in 4 200 ± 800 *τ* (averaged over 8 independent simulations). With increasing number of inhibited RBSs the uptake time was prolonged up to the maximum length of our simulation (50 000 *τ*) at a threshold number.

When the inhibited sites were distributed randomly on the capsid (i.e., mimicking monovalent inhibitors) the threshold for uptake was 24 inhibited sites, which is more than a third of all the RBSs. For monovalent inhibitors, the relationship between bulk concentration and number of bound inhibitors follows the Langmuir adsorption model (see SI). This would mean that to achieve 1/3 fraction coverage we need a bulk mono-inhibitor concentration of *K*/2, where *K* is the binding constant. Typical binding constants in the range from 10^3^ M^−1^ to 10^6^ M^−1^ would lead to high concentrations from 0.5 mM to 0.5 *μ*M that were indeed found necessary in experiments^22,25^.

To mimic the effect of spatially correlated inhibition as can be produced by properly designed multivalent inhibitors, we deactivated the RBSs that were closest to each other (see Fig. 3). The threshold for the uptake was 5 inhibited closest RBSs. This is dramatically smaller than necessary 24 inhibited sites when randomly distributed.

These results show that multivalent inhibitors can be much more effective in hindering the uptake compared to monovalent inhibitors. Already a single multivalent inhibitor bound to the capsid can prevent the uptake as long as more than 4 bindings sites are inhibited. This dramatic improvement, is clearly a co-localization effect and is independent of other classical multivalent properties (i.e., selectivity and affinity) as we need not explicitly modelling the inhibitors.

On top of the co-localization effect, multivalent inhibitors do have a much stronger binding affinity^19,22^ (see Supplementary information), which means that the concentration of multivalent inhibitors in solution necessary to frustrate uptake could be many orders of magnitude smaller than in the monovalent case. That is, it is much more effective to use fewer inhibiting RBSs with individually lower affinity if they are grouped in a multivalent entity (i.e., if they are physically connected) than a large number of independent monovalent inhibitors with high affinity^22,25^. This, together with the potential targeting super-selectivity of the multivalent inhibitors could greatly reduce undesirable side effects while fighting viral infection and has indeed already been observed^25,26^.

**Figure 4:**
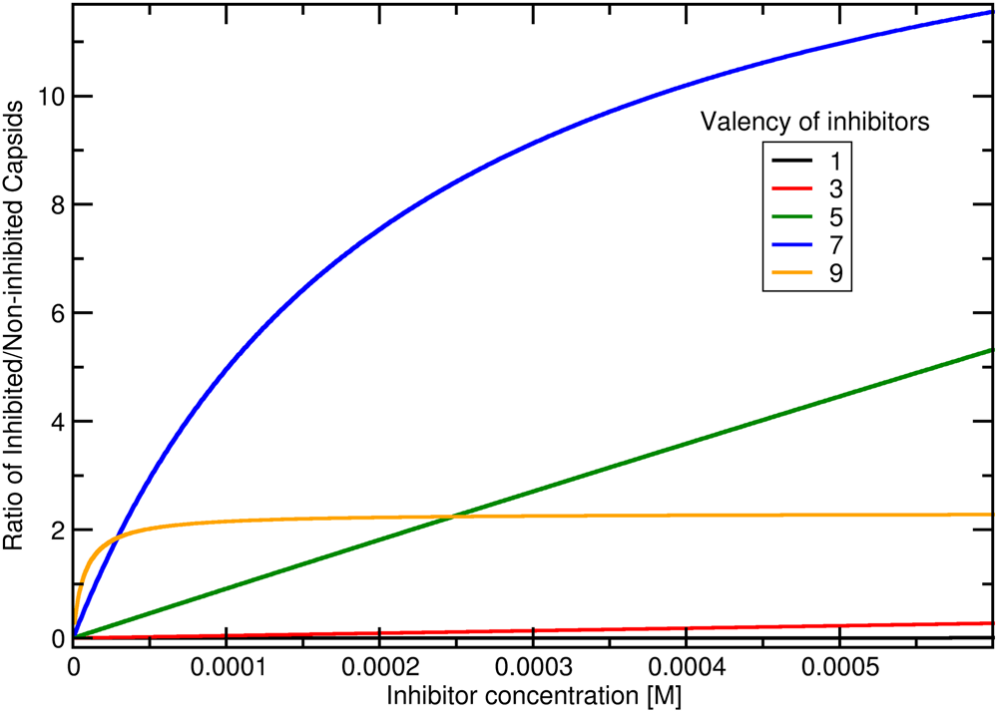
The ratio between inhibited and non-inhibited capsids for various valencies of multivalent inhibitors as a function of the inhibitor concentration. The employed parameters are: *N*_*B*_ = 5 (a multivalent inhibitor can bind to maximum five RBSs on one capsid), *g* = −0.5 kT (the free energy of binding between monovalent inhibitors and the capsid RBSs), and *a*_*C*_ = 0.1 mM (concentration of virus capsids assuming activity coefficient being 1).

A caveat of multivalent inhibitors is that if they are not properly designed they might work as a bridge between viruses causing them to aggregate. Since it has been shown that aggregates of partially inhibited capsids/particles can still be internalized (see Fig. 1D)^26–28^, multivalent inhibitors should be designed to preferentially bind to only one capsid at a time. For example, this could be achieved by designing a Janus multivalent inhibitor. Such Janus inhibitor should have RBSs on only one side, while the other side should be inert, i.e. sterically repel other capsids. Such Janus multivalent inhibitors should also have a decreased removal from blood by macrophages^28^. Other possibility, is to have a limited number of RBSs (i.e., limited valence) per multivalent inhibitor, so the inhibitor would preferentially bind to only one capsid. We derived a simple theory to evaluate ideal multivalent inhibitor binding to either one or two capsids and it can be used to find an optimum valence for multivalent inhibitors (see Fig. 4 and Theory in section Methods). This demonstrates that an increase of multivalency beyond the optimum decreases its inhibition ability, which is in agreement with experimental findings^25^. Note, however, that virus aggregates might also be more effectively removed by macrophages *in vivo*, which has not been considered in our model and would affect the optimal balance. Nevertheless, based on our simulations we would suggest the Janus multivalent inhibitors are the best candidate.

## Conclusion

To summarize, we investigated the process of spontaneous receptor mediated endocytosis where the capsid is closely wrapped by the membrane. We have compared the effect of spontaneous endocytosis inhibition of a model virus by monovalent and multivalent inhibitors. We found that multivalent inhibitors were much more efficient in uptake inhibition. Interestingly, this “super”-inhibition was not only due to the classical multivalent effect of increased affinity, but largely due to the spatially correlated (i.e., localized) inhibition that follows naturally in multivalent entities. To prove this point, we performed simulations with implicit inhibitors in various distributions on the virus surface. We found that when receptor binding sites were inhibited randomly (i.e., mimicking monovalent inhibition), in average 24 or more sites had to be inhibited to prevent uptake. On the other hand, when we were mimicking multivalent inhibition, as little as 5 or 6 binding sites needed to be inhibited to prevent uptake, as long as they were closely localized. This clearly shows that multivalent inhibitors are more efficient not only due to the enhanced cooperative binding strength, but also due to their co-localization of inhibitors. We evaluated the possibility of multivalent inhibitors binding two capsids together and suggested that either valence must be optimized or a Janus shape needs to be used to prevent this effect. Finally, since multivalent inhibitors have the additional advantage that they can be designed to target viruses with super-selective specificity, we expect that they will play a deciding role in the battle against viral infections and nano-medicine in general.

## Methods

### Simulatons

As we are interested in the generic aspects of the inhibition of the uptake process we use a simplified implicit-solvent coarse grained model, in which groups of atoms are represented as beads with effective interactions. Each phospholipid molecule is represented by a chain of three beads^29^. The first bead is a hydrophilic lipid headgroup, which is described with a purely repulsive potential. The second and third beads represent hydrophobic tails and they effectively attract each other with a cosine square potential. The size of beads corresponds roughly to 1 nm. The simplified model receptors were constructed from lipids with a modified headgroup bead, which interacts attractively with beads of the nanoparticles RBSs.

The icosahedral model virus capsid is made of 792 beads leading to a diameter of about 15 nm. The RBSs on the capsid are made of the same beads as lipid beads and they are purely repulsive to all particles except receptor headgroups. This attraction is short ranged (about 0.3 nm) with a −8 kT minimum. The rest of the capsid was formed from purely repulsive beads. The distribution of RBSs was inspired by viruses from the Picornaviridae family (specifically Rhinovirus and Poliovirus), where the RBSs are located between the two fold and five fold axes^30^. The resulting model virus capsid had 60 RBSs made of 120 beads (see Fig. 3). Inside, the capsid was filled with 520 larger beads to keep the icosahedral shape by inner pressure. The explicit inhibitor sites were modeled with the same as beads as the receptor headgroups.

We tested two models for multivalent inhibitors. The first model was a linear polymer of 40 RBS beads. The bond between the beads was the same as between the lipids (FENE bond with stiffness 30 *ε*.*nm* ^−2^ and divergence length 1.5 nm). There was no angular or dihedral potential. The polymer formed a random coil in the solution and a small cluster when bound to the capsid. We used a second model where the overall shape of the multivalent inhibitor was better defined. It was built of 38 beads arranged on hexagonal lattice split into two layers. The choice of two layers was motivated by the ease of conversion of this model to a Janus particle. The neighbouring beads were connected by weak bonds with harmonic shape and stiffness of 3 kT. Our results were independent of the multivalent model, which was further validated with our simulations wherein the multivalent inhibitor was implicitly modelled by inactivated RBSs on the capsid. Thus, showing that the nature of the colocalized inhibition phenomenon is largely independent of the specifics of the model. For the Janus inhibitor, we used only the two-layer model, where one layer was built from purely repulsive beads with the size of lipid beads.

The model bilayer was created from 8000 phospholipid molecules placed in a rectangular box with periodic boundary conditions. Half of the lipids were modified to become receptors. The excess of receptors was chosen to eliminate the effects of receptor diffusion. The membrane was kept at zero tension and Molecular Dynamics was performed with the program ESPRESSO^31^. The Langevin thermostat kept the system temperature at 1.0 kT, where the membrane is in the liquid fluid phase^29^. The assumption of a tension-less membrane is valid for the cellular uptake of nano-objects smaller than a few hundred nanometers^32^.

The models and parameters chosen have been widely used in previous studies of uptake of nano-objects affected by their size, shape, total number of RBSs, strength of the RBS-receptor interaction, and spatial distribution of RBSs^3,12,33^. The simulation time unit *τ* can be related to 100 ns based on the lipid diffusion; however, this should be considered as a very rough estimate since hydrodynamic effects are missing in our implicit solvent model. More details about the model and the effects of other parameters (size, strength of the RBS-receptor interaction, etc.) on the uptake can be found in refs^3,12,33^.

The time of the uptake was evaluated from the snapshots and from the time dependence of the box size and the receptor-RBS interaction energy, which have been shown well suited to investigate the uptake time and presence of possible metastable states^12^. Langevin termostat on constant level with the set up of friction constant gama = 1.0 τ^−1^. Barostat provided the constant pressure 0.000 kg × m-1 × tau-2 in xy plane with weight of the piston 0.0005. The time step was set up on 0.001 τ.

Multivalent inhibitors can lead to attraction and binding of two ore more virus capsids. We constructed a model system with multivalent inhibitor represented as simple chain of beads attracted to the receptor binding sites (RBS). The two capsids readily bound to each other and together were uptaken in less than 4 000 *τ* (see Fig. 1C). The change of the orientation of the capsid to the membrane change during the uptake as described previously for elongated nanoparticles^3,34^.

### Theory

We can estimate the binding of the a multivalent inhibitor to a capsid and binding of two capsids together by the inhibitor by an analytical model^19^. The assumptions are that [1] inhibitors and RBSs do not affect each other except through the inhibitor-RBS binding [2] individual inhibitor parts are ideal, i.e. not affecting each other except by the occupation of the RBS. The bound state partition function is then given by:

1 $${q}_{1C}=\sum _{s=1}^{{\rm{\min }}({N}_{I},{N}_{B})}Q(s)={e}^{-{\rm{\Delta }}{G}_{1C}/kT}$$

where *N*_*I*_ is the valency of the multivalent inhibitor, *N*_*B*_ is the number of available RBSs to one bound multivalent inhibitor, *G*_1*C*_ is the binding free energy between the capsid and one multivalent inhibitor, and *Q*(*s*) is the partition sum for realizations of *s* bonds between single multivalent inhibitor and a capsid given by ref.^19^:

2 $$Q(s)=\frac{{N}_{I}!{N}_{B}!}{({N}_{I}-s)!s!({N}_{B}-s)!}{e}^{-gs/kT}$$

*g* is the free energy of monovalent inhibitor binding to RBS. The probability of the bound multivalent inhibitor to have exactly *s* bonds, *P*(*s*), is $P(s)=\frac{Q(s)}{{q}_{1C}}$ and can be evaluated by the attached script.

Similarly, we can derive an expression for one inhibitor binding two capsids. The partition sum for a realization of the inhibitor binding one capsid with *s* bonds and binding the second capsid with *t* bonds is:

3 $$Q(s,t)=\frac{{N}_{I}!{N}_{B}!{N}_{B}!\exp [-g(s+t)/kT]}{({N}_{I}-s-t)!s!t!({N}_{B}-s)!({N}_{B}-t)!}$$

The bound state partition function of two capsid is then:

4 $${q}_{2C}=\sum _{s=1}^{{\rm{\min }}({N}_{I},{N}_{B})}\sum _{t=1}^{{\rm{\min }}({N}_{I},{N}_{B})}Q(s,t)={e}^{-{\rm{\Delta }}{G}_{2C}/kT}$$

where *s* + *t* ≤ min(*N*_*I*_, 2*N*_*B*_) and *G*_2*C*_ is the binding free energy of two capsids by a multivalent inhibitor.

Using this analytical model, we can calculate the fraction of capsids with one bound inhibitor, i.e. capsids inhibited from uptake, ${\varphi }_{1}=\frac{{N}_{inhibited}}{{N}_{withoutinhibitor}}$. ${\varphi }_{2}=\frac{{N}_{2capsids}}{{N}_{withoutinhibitor}}$, the fraction of capsids dimers, i.e. capsids bound together by a multivalent inhibitor inhibitor (Fig. 2B), are not inhibited for uptake can be calculated as:

5 $$\begin{array}{cc}{\varphi }_{1}={q}_{1C}\frac{{a}_{I}}{{c}^{o}} & {\varphi }_{2}={q}_{2C}\frac{{a}_{C}{a}_{I}}{{c}^{o2}}\end{array}$$

where *a*_*C*_ and *a*_*I*_ are the activities of the capsids and inhibitors respectively. Activities are related to concentrations via activity coefficients and at low concentrations the activities are equal to concentrations. *c* ^*o*^ is the standard concentration.

The ratio between inhibited and non-inhibited capsids is $\frac{{N}_{inhibited}}{{N}_{withoutinhibitor}+{N}_{2capsids}}$:

6 $$\frac{{\varphi }_{1}}{{\varphi }_{2}+1}=\frac{{q}_{1C}{a}_{I}{c}^{o}}{{q}_{2C}{a}_{C}{a}_{I}+{c}^{o2}}$$

Here we assume that capsid with a bound inhibitor is already inhibited and the inhibitor concentration is low, so we can neglect the number of capsids with more than one inhibitor. Moreover, larger aggregates are neglected and the system is in equilibrium.

For practical purposes, we calculated dimensionless binding constant of one inhibitor to one (1C) or two (2C) capsids for various parameters of *N*_*I*_, *N*_*B*_, and *g*, which can be found together with a script to calculate those in Supplementary information. Figure 4 captures fraction of inhibited capsids and demonstrates that due to the possibility of binding more than one capsid the multivalent inhibitors have a valency that is the most effective. In Fig. 4 the optimum valency is 7, however, this optimum is dependent on the particular system and a small change can result in a different optimal valency. For example, the change of monomer binding strength, *g*, from −0.5 kT to −3.5 kT (keeping *N*_*B*_ and *a*_*C*_ the same) shifts the optimum valency from 7 to 3. Regardless of details, however, multivalent inhibitors stay more effective than monovalent ones.

## Electronic supplementary material

Supplementary Information

Python script for calculation of bining constants

Pythons script for evaluation of inhibited sites
