# Supplementary material for: Design of Multivalent Inhibitors for Preventing Cellular Uptake
Source: Sci Rep. 2017 Sep 15;7:11689. doi: 10.1038/s41598-017-11735-7 (PMC5601900; doi:10.1038/s41598-017-11735-7)
Supplement: Supplementary file 1 — Supplementary Information [file 41598_2017_11735_MOESM1_ESM.pdf]

Design of Multivalent Inhibitors for Preventing Cellular Uptake

Veronika Schubertová[1], Francisco J. Martinez-Veracoechea[2], and Robert Vácha[1,*]

[1]Faculty of Science and CEITEC, Masaryk University, Kamenice 5, 625 00 Brno, Czech Republic
[2]Eesti Energia AS, Lelle 22, 11318 Tallinn, Estonia
[*]robert.vacha@mail.muni.cz,robertvacha@gmail.com

ABSTRACT

Supplementary Information

Uptake for capsids with various inhibitors

The uptake process can be captured by changes in the interaction energy between receptors and receptor binding sites (RBSs). Similarly, the uptake can be described by changes in the simulation box size in membrane plane, which is related to the amount of membrane wrapping. Figures S1 - S5 depicts the simulations with a virus-like capsid without inhibitor, with multivalent inhibitor, with Janus multivalent inhibitor, with many monovalent inhibitors, and with second capsid interacting via multivalent inhibitor respectively. Occasional metastable states were in most cases related to necessary membrane fluctuation, capsid rotation, or the desorption of monomer inhibitors.

Note that in case of monovalent inhibitors, the strong inhibitor binding to the capsid effectively decreases the concentration of free monomers in solution. This is caused by the fixed number of particles in the simulation and the limited size of simulation box. In reality, additional monomers would diffuse to the location from bulk solution. To be able to relate to bulk concentrations we performed additional simulations without membrane, where we investigated the average number of inhibitors in solution and at the capsid (see Tables below).

To clarify the effect of monovalent inhibitors on the uptake, we performed simulations with a different number of monovalent inhibitors in the system and different inhibitor-RBS interaction strength. As expected, Figure S4 shows that the uptake gets slower with increasing number and binding of monovalent inhibitors. During the uptake, some of the inhibitors remained bound to the capsid, but most of them desorb. The number of inhibitors in solution is changing during the simulations, which makes it difficult to compare to experiments with particular concentration. Moreover, the observed uptake is likely to be affected by the size of the monovalent inhibitors. All these effects make the uptake very dependent on the model. Therefore, we constructed and focused on the idealized model of monovalent very strong inhibitors, which inhibit RBS but have no size and remained adsorbed.

Table S1. Average number of monovalent inhibitors interacting with model capsid (Inhibitor-RBS= -8 kT) for given concentration. Estimated remaining bulk concentration in box is also displayed.

Box concentration/mM	# Inhibitors in box	# Bound inhibitors	Free concentration/mM
0.05	10	10	0
0.1	25	24	0.005
0.3	50	47	0.015
0.5	100	91	0.05

Table S2. Average number of monovalent inhibitors interacting with model capsid (Inhibitor-RBS= -5 kT) for given concentration. Estimated remaining bulk concentration in box is also displayed.

Box concentration/mM	# Inhibitors in box	# Bound inhibitors	Free concentration/mM
0.3	50	7	0.2
0.5	100	10	0.5
1.0	200	21	1.0
2.0	400	36	2.0

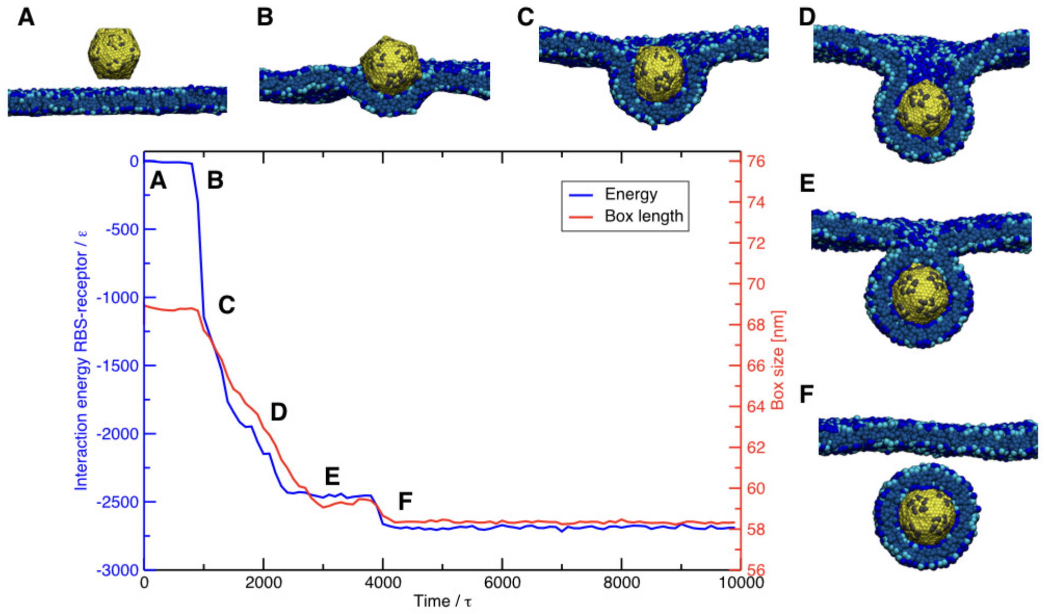

Figure S1. Interaction energy between RBS and receptors and box length as a function of simulation time demonstrates the uptake path and uptake time of the virus capsid. Illustrative snapshots of virus like capsid with 60 RBSs at different times during the uptake. Color coding: Capsid - yellow represent hydrophilic beads, while gray highlights binding sites. Phospholipid molecules are shown blue (hydrophilic parts – dark blue, membrane receptors – light blue, and hydrophobic part – mid blue)

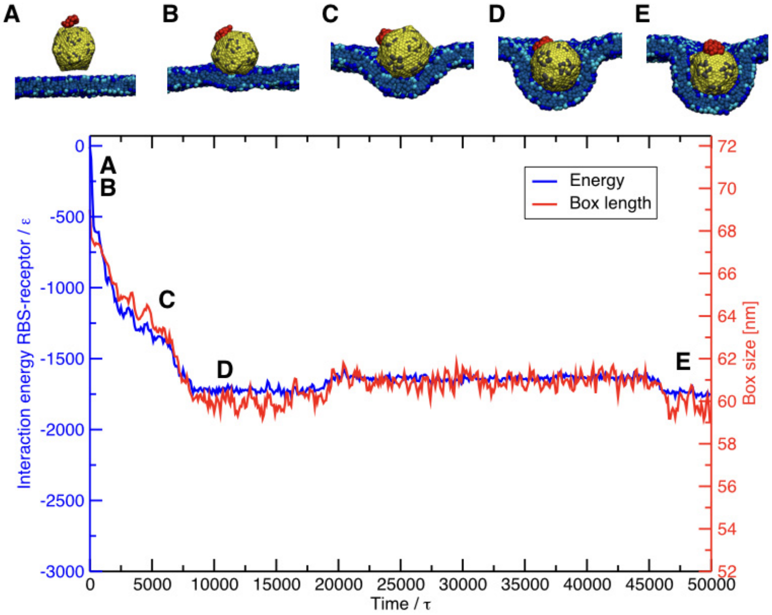

Figure S2. Interaction energy between RBS and receptors and box length as a function of simulation time demonstrates the uptake path and uptake time of the virus capsid in presence of multivalent inhibitor. Illustrative snapshots of virus like capsid with 60 RBSs and multivalent inhibitor at different times during the uptake. Color coding: Capsid - yellow represent hydrophilic beads, while gray highlights binding sites. Inhibitors - red. Phospholipid molecules are shown blue (hydrophilic parts – dark blue, membrane receptors – light blue, and hydrophobic part – mid blue)

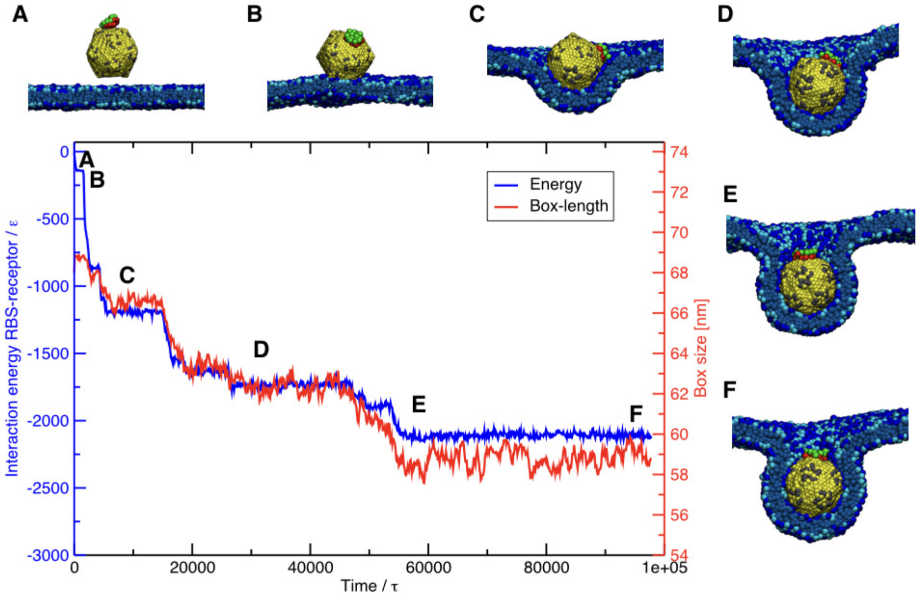

Figure S3. Interaction energy between RBS and receptors and box length as a function of simulation time demonstrates the uptake path and uptake time of the virus capsid in presence of Janus inhibitor. Illustrative snapshots of virus like capsid with 60 RBSs and Janus inhibitor at different times during the uptake. Color coding: Capsid - yellow represent hydrophilic beads, while gray highlights binding sites. Inhibitors - red, with non interacting part in green. Phospholipid molecules are shown blue (hydrophilic parts – dark blue, membrane receptors – light blue, and hydrophobic part – mid blue)

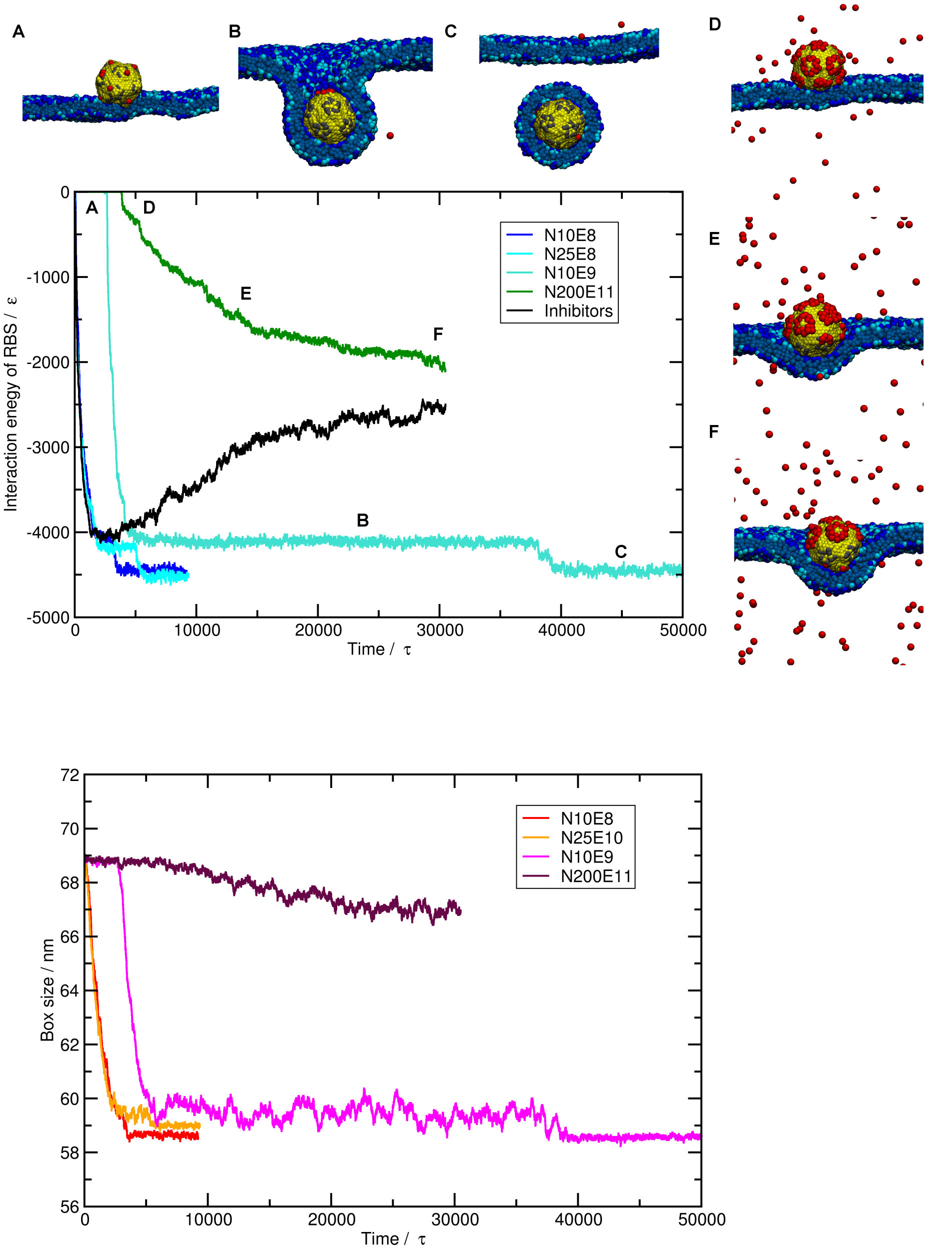

Figure S4. Interaction energy between RBS and receptors (UPPER) and box length (LOWER) as a function of simulation time demonstrates the uptake path and uptake time of the virus capsid in presence of monovalent inhibitors at various inhibitor-RBS binding strengths (E = -8.0, -9.0, or -11.0 kT) and number of inhibitors in the system (N = 10, 25, or 200). Interaction energy between the capsid and inhibitors is depicted for system N100E11, which shows the desorption of inhibitors during the uptake.

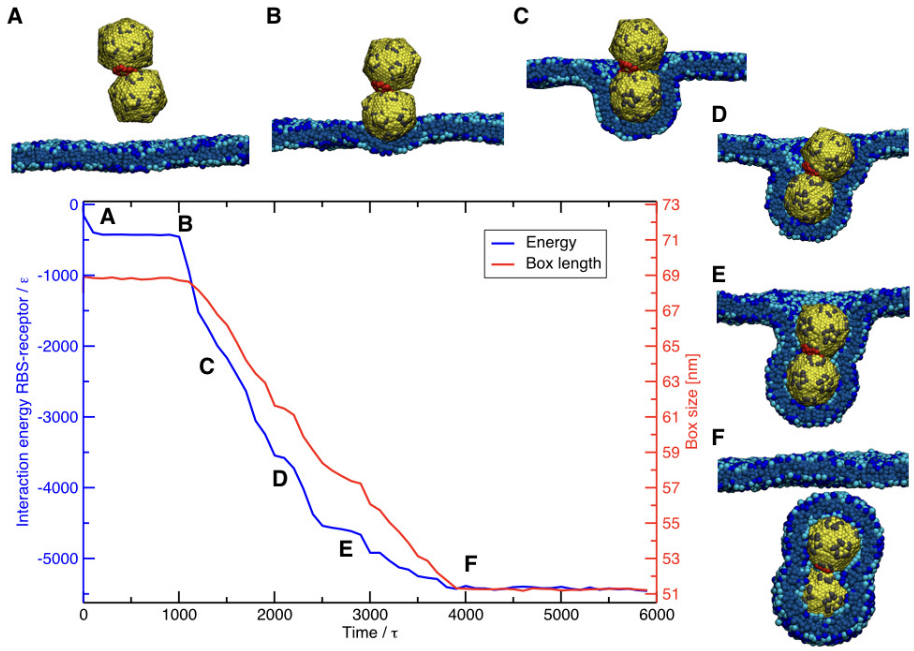

Figure S5. Interaction energy between RBS and receptors and box length as a function of simulation time demonstrates the uptake path and uptake time of the two virus capsid in presence of multivalent inhibitor (polymer model). Illustrative snapshots of virus like capsid with 60 RBSs and multivalent inhibitor at different times during the uptake. Color coding: Capsid - yellow represent hydrophilic beads, while gray highlights binding sites. Inhibitors - red. Phospholipid molecules are shown blue (hydrophilic parts – dark blue, membrane receptors – light blue, and hydrophobic part – mid blue)

Randomly distributed inhibited binding sites

When monovalent inhibitors bind to the capsid without affecting each other (Langmuir model), they are randomly distributed. We have evaluated the probability of such random distributions to inhibit specific geometries, e.g. five closest receptor binding sites (RBS). The results are in the following table. Because we have seen the inhibition at 24 randomly inhibited sites it is from the table clear that it is not necessary to inhibit all five closest binding sites but also more spread distributions are also able to inhibit the uptake. Indeed, in our simulations with 24 inhibited binding sites on the capsid only three out of five were inhibited but there were other inhibited sites near by.

Table S3. Probability that randomly distributed inhibited binding sites will occupy at least four or five closest binding sites. The geometries of four closest binding sites is considered the same as in Figure 2 - geometry RBS56 for four and RBS55 for five closest RBSs.

Number of randomly inhibited sites	Probability inhibit four closest - RBS56	Probability inhibit five closest - RBS55	Probability inhibit four out of RBS55	Probability inhibit three out of RBS55
4	0.0001	0.0000	0.0000	0.002
5	0.0006	0.0000	0.0002	0.005
6	0.0019	0.0000	0.0004	0.010
7	0.0044	0.0000	0.001	0.017
8	0.0087	0.0000	0.002	0.027
9	0.0155	0.0001	0.003	0.038
10	0.025	0.0002	0.005	0.052
11	0.040	0.0005	0.008	0.069
12	0.060	0.0008	0.012	0.088
13	0.084	0.001	0.017	0.109
14	0.116	0.002	0.023	0.133
15	0.155	0.003	0.030	0.159
16	0.201	0.004	0.038	0.186
17	0.255	0.006	0.048	0.216
18	0.315	0.008	0.060	0.247
19	0.381	0.010	0.073	0.280
20	0.451	0.013	0.089	0.314
21	0.525	0.017	0.106	0.348
22	0.598	0.021	0.124	0.383
23	0.670	0.027	0.145	0.420
24	0.738	0.033	0.167	0.455
25	0.799	0.041	0.191	0.492
26	0.852	0.050	0.217	0.528
27	0.897	0.059	0.244	0.563
28	0.931	0.070	0.273	0.598
29	0.957	0.083	0.304	0.631
30	0.975	0.097	0.335	0.665
31	0.986	0.113	0.369	0.697
32	0.993	0.131	0.402	0.726
33	0.997	0.150	0.437	0.755
34	0.999	0.171	0.472	0.784

The number of inhibited(bound) sites, $P(s)$:

NB = number of available receptor binding sites for multivalent inhibitor on each capsid, N_I = valency of multivalent inhibitor, and g = binding free energy per monomer (g = -10 kT corresponds to K = 22 $\times 10^3$ M; g = -1 kT corresponds to K = 2.7 M; g = -0.1 kT corresponds to K = 1.1 M; g = -0.01 kT corresponds to K = 1.01 M; g = -0.001 kT corresponds to K = 1.001 M) Our python script that calculates the probability is part of the supplementary information.

Table S4. $N_B = 4; N_I = 5$

s \ g	-10	-1	-0.1	-0.01	-0.001
1	1.56e-14	4.42e-03	3.29e-02	3.92e-02	3.99e-02
2	2.06e-09	7.20e-02	2.18e-01	2.38e-01	2.40e-01
3	9.08e-05	3.91e-01	4.82e-01	4.80e-01	4.80e-01
4	1.00e+00	5.32e-01	2.67e-01	2.43e-01	2.40e-01

Table S5. $N_B = 6; N_I = 5$

s \ g	-10	-1	-0.1	-0.01	-0.001
1	1.77e-19	3.52e-04	5.63e-03	7.21e-03	7.39e-03
2	3.90e-14	9.57e-03	6.22e-02	7.28e-02	7.39e-02
3	3.43e-09	1.04e-01	2.75e-01	2.94e-01	2.96e-01
4	1.13e-04	4.24e-01	4.56e-01	4.46e-01	4.45e-01
5	1.00e+00	4.62e-01	2.01e-01	1.80e-01	1.78e-01

Table S6. $N_B = 4; N_I = 7$

s \ g	-10	-1	-0.1	-0.01	-0.001
1	3.12e-15	1.18e-03	1.14e-02	1.40e-02	1.43e-02
2	6.18e-10	2.88e-02	1.13e-01	1.27e-01	1.28e-01
3	4.54e-05	2.61e-01	4.16e-01	4.27e-01	4.28e-01
4	1.00e+00	7.09e-01	4.60e-01	4.32e-01	4.29e-01

Table S7. $N_B = 6; N_I = 7$

s \ g	-10	-1	-0.1	-0.01	-0.001
1	1.61e-24	2.26e-05	7.82e-04	1.08e-03	1.11e-03
2	5.31e-19	9.21e-04	1.30e-02	1.63e-02	1.67e-02
3	7.80e-14	1.67e-02	9.55e-02	1.10e-01	1.11e-01
4	5.15e-09	1.36e-01	3.16e-01	3.33e-01	3.35e-01
5	1.36e-04	4.44e-01	4.20e-01	4.04e-01	4.02e-01
6	1.00e+00	4.02e-01	1.55e-01	1.36e-01	1.34e-01

The bound state partition function of one capsid, q_1C:

NB = number of available receptor binding sites for multivalent inhibitor on each capsid, N_I = valency of multivalent inhibitor, and g = binding free energy per monomer (g = -10 kT corresponds to K = 22 $\times 10^3$ M; g = -1 kT corresponds to K = 2.7 M; g = -0.1 kT corresponds to K = 1.1 M; g = -0.01 kT corresponds to K = 1.01 M; g = -0.001 kT corresponds to K = 1.001 M) Our python script that calculates the binding constants is part of the supplementary information.

Table S8. N_B=3

N_I \ g/kT	-10	-1	-0.1	-0.01	-0.001
1	6.61e+04	8.15e+00	3.32e+00	3.03e+00	3.00e+00
2	2.91e+09	6.06e+01	1.40e+01	1.22e+01	1.20e+01
3	6.41e+13	2.78e+02	4.00e+01	3.36e+01	3.31e+01
4	2.56e+14	7.81e+02	8.96e+01	7.36e+01	7.22e+01
5	6.41e+14	1.69e+03	1.71e+02	1.38e+02	1.35e+02
6	1.28e+15	3.12e+03	2.92e+02	2.34e+02	2.29e+02
7	2.24e+15	5.21e+03	4.61e+02	3.66e+02	3.58e+02
8	3.59e+15	8.06e+03	6.85e+02	5.42e+02	5.29e+02
9	5.39e+15	1.18e+04	9.74e+02	7.67e+02	7.49e+02
10	7.69e+15	1.65e+04	1.33e+03	1.05e+03	1.02e+03
11	1.06e+16	2.24e+04	1.78e+03	1.39e+03	1.36e+03
12	1.41e+16	2.95e+04	2.31e+03	1.80e+03	1.76e+03
13	1.83e+16	3.80e+04	2.93e+03	2.29e+03	2.23e+03
14	2.33e+16	4.80e+04	3.66e+03	2.85e+03	2.78e+03

Table S9. N_B=5

N_I \ g/kT	-10	-1	-0.1	-0.01	-0.001
1	1.10e+05	1.36e+01	5.53e+00	5.05e+00	5.01e+00
2	9.70e+09	1.75e+02	3.55e+01	3.05e+01	3.01e+01
3	6.41e+14	1.69e+03	1.71e+02	1.38e+02	1.35e+02
4	2.82e+19	1.23e+04	6.72e+02	5.15e+02	5.01e+02
5	6.22e+23	6.42e+04	2.17e+03	1.60e+03	1.55e+03
6	3.73e+24	2.32e+05	5.89e+03	4.20e+03	4.07e+03
7	1.31e+25	6.49e+05	1.38e+04	9.65e+03	9.31e+03
8	3.48e+25	1.53e+06	2.89e+04	1.99e+04	1.92e+04
9	7.84e+25	3.18e+06	5.52e+04	3.76e+04	3.62e+04
10	1.57e+26	6.02e+06	9.83e+04	6.64e+04	6.39e+04
11	2.87e+26	1.06e+07	1.65e+05	1.11e+05	1.07e+05
12	4.93e+26	1.76e+07	2.65e+05	1.77e+05	1.70e+05
13	8.01e+26	2.80e+07	4.08e+05	2.71e+05	2.60e+05
14	1.25e+27	4.27e+07	6.07e+05	4.02e+05	3.86e+05

Table S10. N_B=6

N_I \ g/kT	-10	-1	-0.1	-0.01	-0.001
1	1.32e+05	1.63e+01	6.63e+00	6.06e+00	6.01e+00
2	1.46e+10	2.54e+02	4.99e+01	4.27e+01	4.21e+01
3	1.28e+15	3.12e+03	2.92e+02	2.34e+02	2.29e+02
4	8.47e+19	3.07e+04	1.43e+03	1.08e+03	1.05e+03
5	3.73e+24	2.32e+05	5.89e+03	4.20e+03	4.07e+03
6	8.22e+28	1.28e+06	2.03e+04	1.39e+04	1.34e+04
7	5.76e+29	5.05e+06	5.94e+04	3.94e+04	3.78e+04
8	2.30e+30	1.56e+07	1.51e+05	9.79e+04	9.37e+04
9	6.91e+30	4.06e+07	3.42e+05	2.18e+05	2.09e+05
10	1.73e+31	9.24e+07	7.09e+05	4.46e+05	4.26e+05
11	3.80e+31	1.90e+08	1.36e+06	8.49e+05	8.10e+05
12	7.60e+31	3.63e+08	2.46e+06	1.52e+06	1.45e+06
13	1.41e+32	6.51e+08	4.21e+06	2.59e+06	2.47e+06
14	2.47e+32	1.11e+09	6.92e+06	4.23e+06	4.03e+06

Table S11. N_B=10

N_I \ g/kT	-10	-1	-0.1	-0.01	-0.001
1	2.20e+05	2.72e+01	1.11e+01	1.01e+01	1.00e+01
2	4.37e+10	7.19e+02	1.32e+02	1.12e+02	1.10e+02
3	7.69e+15	1.65e+04	1.33e+03	1.05e+03	1.02e+03
4	1.19e+21	3.37e+05	1.21e+04	8.80e+03	8.53e+03
5	1.57e+26	6.02e+06	9.83e+04	6.64e+04	6.39e+04
6	1.73e+31	9.24e+07	7.09e+05	4.46e+05	4.26e+05
7	1.52e+36	1.19e+09	4.49e+06	2.65e+06	2.52e+06
8	1.01e+41	1.27e+10	2.49e+07	1.38e+07	1.31e+07
9	4.43e+45	1.08e+11	1.20e+08	6.32e+07	5.94e+07
10	9.76e+49	7.11e+11	4.99e+08	2.53e+08	2.36e+08
11	1.07e+51	3.64e+12	1.82e+09	8.92e+08	8.31e+08
12	6.44e+51	1.50e+13	5.88e+09	2.80e+09	2.60e+09
13	2.79e+52	5.21e+13	1.70e+10	7.95e+09	7.37e+09
14	9.77e+52	1.58e+14	4.50e+10	2.06e+10	1.91e+10

The bound state partition function of two capsids, q_2C:

NB = number of available receptor binding sites for multivalent inhibitor on each capsid, g = binding energy per monomer, N_I = valency of multivalent inhibitor Our python script that calculates the binding constants is part of the supplementary information.

Table S12. N_B=3

N_I \ g/kT	-10	-1	-0.1	-0.01	-0.001
1	0.00e+00	0.00e+00	0.00e+00	0.00e+00	0.00e+00
2	8.73e+09	1.33e+02	2.20e+01	1.84e+01	1.80e+01
3	1.15e+15	2.57e+03	2.12e+02	1.66e+02	1.62e+02
4	5.08e+19	2.13e+04	1.04e+03	7.80e+02	7.58e+02
5	2.54e+20	8.20e+04	3.29e+03	2.42e+03	2.35e+03
6	7.63e+20	2.22e+05	8.08e+03	5.87e+03	5.69e+03
7	1.78e+21	4.91e+05	1.68e+04	1.21e+04	1.18e+04
8	3.56e+21	9.51e+05	3.13e+04	2.25e+04	2.18e+04
9	6.41e+21	1.67e+06	5.36e+04	3.83e+04	3.71e+04
10	1.07e+22	2.74e+06	8.62e+04	6.14e+04	5.94e+04
11	1.68e+22	4.26e+06	1.32e+05	9.36e+04	9.04e+04
12	2.52e+22	6.32e+06	1.93e+05	1.37e+05	1.32e+05
13	3.64e+22	9.06e+06	2.74e+05	1.94e+05	1.87e+05
14	5.09e+22	1.26e+07	3.78e+05	2.67e+05	2.58e+05

Table S13. N_B=5

N_I \ g/kT	-10	-1	-0.1	-0.01	-0.001
1	0.00e+00	0.00e+00	0.00e+00	0.00e+00	0.00e+00
2	2.43e+10	3.69e+02	6.11e+01	5.10e+01	5.01e+01
3	6.41e+15	1.32e+04	9.93e+02	7.71e+02	7.52e+02
4	1.13e+21	3.12e+05	1.08e+04	7.78e+03	7.53e+03
5	1.56e+26	5.89e+06	9.40e+04	6.32e+04	6.08e+04
6	1.64e+31	8.90e+07	6.84e+05	4.30e+05	4.11e+05
7	1.27e+36	1.06e+09	4.17e+06	2.47e+06	2.35e+06
8	5.59e+40	9.32e+09	2.10e+07	1.18e+07	1.12e+07
9	5.03e+41	5.24e+10	8.60e+07	4.68e+07	4.40e+07
10	2.51e+42	2.15e+11	2.92e+08	1.55e+08	1.46e+08
11	9.22e+42	7.07e+11	8.52e+08	4.46e+08	4.18e+08
12	2.76e+43	1.98e+12	2.20e+09	1.14e+09	1.07e+09
13	7.19e+43	4.92e+12	5.15e+09	2.64e+09	2.47e+09
14	1.68e+44	1.11e+13	1.11e+10	5.66e+09	5.29e+09

Table S14. N_B=6

N_I \ g/kT	-10	-1	-0.1	-0.01	-0.001
1	0.00e+00	0.00e+00	0.00e+00	0.00e+00	0.00e+00
2	3.49e+10	5.32e+02	8.79e+01	7.35e+01	7.21e+01
3	1.15e+16	2.33e+04	1.72e+03	1.33e+03	1.30e+03
4	2.63e+21	6.99e+05	2.30e+04	1.65e+04	1.60e+04
5	4.85e+26	1.72e+07	2.53e+05	1.68e+05	1.62e+05
6	7.58e+31	3.61e+08	2.42e+06	1.49e+06	1.42e+06
7	9.89e+36	6.50e+09	2.03e+07	1.17e+07	1.10e+07
8	1.04e+42	9.87e+10	1.49e+08	8.02e+07	7.54e+07
9	7.97e+46	1.21e+12	9.44e+08	4.79e+08	4.48e+08
10	3.51e+51	1.13e+13	5.09e+09	2.45e+09	2.28e+09
11	3.86e+52	7.15e+13	2.29e+10	1.06e+10	9.86e+09
12	2.32e+53	3.38e+14	8.70e+10	3.94e+10	3.64e+10
13	1.00e+54	1.28e+15	2.86e+11	1.27e+11	1.17e+11
14	3.52e+54	4.12e+15	8.34e+11	3.65e+11	3.37e+11

Table S15. N_B=10

N_I \ g/kT	-10	-1	-0.1	-0.01	-0.001
1	0.00e+00	0.00e+00	0.00e+00	0.00e+00	0.00e+00
2	9.70e+10	1.48e+03	2.44e+02	2.04e+02	2.00e+02
3	5.77e+16	1.13e+05	8.02e+03	6.18e+03	6.02e+03
4	2.50e+22	6.24e+06	1.89e+05	1.34e+05	1.29e+05
5	9.33e+27	2.97e+08	3.84e+06	2.50e+06	2.40e+06
6	3.15e+33	1.28e+10	7.06e+07	4.24e+07	4.03e+07
7	9.80e+38	5.11e+11	1.20e+09	6.67e+08	6.29e+08
8	2.81e+44	1.89e+13	1.92e+10	9.77e+09	9.14e+09
9	7.44e+49	6.46e+14	2.84e+11	1.34e+11	1.24e+11
10	1.80e+55	2.04e+16	3.94e+12	1.71e+12	1.58e+12
11	3.97e+60	5.94e+17	5.06e+13	2.04e+13	1.86e+13
12	7.86e+65	1.57e+19	6.01e+14	2.24e+14	2.03e+14
13	1.38e+71	3.76e+20	6.58e+15	2.28e+15	2.05e+15
14	2.12e+76	8.06e+21	6.60e+16	2.13e+16	1.91e+16
