# Supplementary material for: Design of Multivalent Inhibitors for Preventing Cellular Uptake
Source: Sci Rep. 2017 Sep 15;7:11689. doi: 10.1038/s41598-017-11735-7 (PMC5601900; doi:10.1038/s41598-017-11735-7)
Supplement: Supplementary file 2 — Python script for calculation of bining constants [file 41598_2017_11735_MOESM2_ESM.pdf]

```
#!/usr/bin/env python

import optparse
import math
from math import factorial
import sys

def do_combinationsC1(NB,NI,g):
    binding1=0
    for i in range(1,NB+1):
      if (i <= NI):
          comb1=factorial(NI)/factorial(NI-i)*factorial(NB)/factorial(NB-
i)/factorial(i)
          binding1=binding1+comb1*math.exp(-g*i)
    return binding1

def do_combinationsC2(NB,NI,g):
    binding2=0
    weights=[]
    for i in range(0,2*NB+1):
      weights.append(0)
    for i in range(1,NB):
      for j in range (1,NB):
          if (i+j <= NI):
            comb2=factorial(NI)/factorial(NI-i-j)*factorial(NB)/factorial(NB-
i)*factorial(NB)/factorial(NB-j)/factorial(i)/factorial(j)
            weights[i+j]+=comb2
    for i in range(2,2*NB+1):
      binding2=binding2+weights[i]*math.exp(-g*i)
    return binding2

def do_bindingconstants(NImax,NBs,gs):
    print "# q1C(bound state partition function to one capsid)"
    for NB in NBs:
#     print "\n\nNB=", NB, "# number of available binding sites for multivalent
inhibitor on each capsid"
#     print " #top g(bingind energy per monomer) left side N_I(#of inhibitors on
multi-inhibitor)"
      print "\n\nNB=", NB
      print "\\begin{table}[] \n \\centering \n \\begin{tabular}{|c||c|c|c|c|c|}
\n \\hline \n \\diaghead{NIgaaaa}{$N_I$}{$g$} ",
      for g in gs:
#         print "\t",g,
          print " & ", "\t",g,
      print "\\\\ \\hline \\hline",
      for NI in range(1,NImax):
          print "\n %2d" % NI,
          for g in gs:
            binding1 = do_combinationsC1(NB,NI,g)
#           print "%8.2e"%binding1 ,
            print " & ", "%8.2e"%binding1 ,
          print "\\\\ \\hline",
      print "\n \\end{tabular} \n \\end{table}"

    print "# q2C(bound state partition function to two capsids)"
    for NB in NBs:
#     print "\n\nNB=", NB, "# number of available binding sites for multivalent
inhibitor on each capsid"
#     print " #top g(bingind energy per monomer) left side N_I(#of inhibitors on
multi-inhibitor)"
```

```
      print "\n\nNB=", NB
      print "\\begin{table}[] \n \\centering \n \\begin{tabular}{|c||c|c|c|c|c|}
\n \\hline \n \\diaghead{NIgaaaa}{$N_I$}{$g$} ",
      for g in gs:
#         print "\t",g,
          print " & ", "\t",g,
      print "\\\\ \\hline \\hline",
      for NI in range(1,NImax):
          print "\n %2d" % NI,
          for g in gs:
            binding2 = do_combinationsC2(NB,NI,g)
#           print "%8.2e"%binding2 ,
            print  " & ", "%8.2e"%binding2 ,
          print "\\\\ \hline",
      print "\n \\end{tabular} \n \\end{table}"


parser=optparse.OptionParser()
help="""Usage:
%prog [options].
"""
parser.set_usage(help)
parser.add_option(
    "--NImax",
    help="Maximum valency of multivalent inhibitor",
    dest="NImax",
    default=15
    )
parser.add_option(
    "--NB",
    help="Number of avaible binding sites on one capsid for one bound
multivalent inhibitor (e.g. [3,5,6,10])",
    dest="NBs",
    default=[3,5,6,10]
    )
parser.add_option(
    "--g",
    help="Binding free energy of only monovalnet inhibitor to one binding site",
    dest="gs",
    default=[-10,-1,-0.1,-0.01,-0.001]
    )

(options,arguments)=parser.parse_args()
do_bindingconstants(options.NImax,options.NBs,options.gs)
```
