# Supplementary material for: Design of Multivalent Inhibitors for Preventing Cellular Uptake
Source: Sci Rep. 2017 Sep 15;7:11689. doi: 10.1038/s41598-017-11735-7 (PMC5601900; doi:10.1038/s41598-017-11735-7)
Supplement: Supplementary file 3 — Pythons script for evaluation of inhibited sites [file 41598_2017_11735_MOESM3_ESM.pdf]

```
#!/usr/bin/env python

import optparse
import math
from math import factorial
import sys

def do_Qs(NB,NI,g,s):
    return factorial(NI)/factorial(NI-s)*factorial(NB)/factorial(NB-
s)/factorial(s)*math.exp(-g*s)

def do_q1C(NB,NI,g):
    q1C=0
    for i in range(1,NB+1):
      if (i <= NI):
          q1C+=do_Qs(NB,NI,g,i)
#         print "q1C is:",  "%8.6e"%q1C
    return q1C

def do_probab(NI,NBs,gs):
    print "# Probability of number of inhibited sites"
    for NB in NBs:
#     print "\n\nNB=", NB, "# number of available binding sites for multivalent
inhibitor on capsid"
#     print " #top g(bingind energy per monomer) left side s(#of inhibited
sites)"
      print "\n\nNB =", NB, "; NI =", NI
      print "\\begin{table}[] \n \\centering \n \\begin{tabular}{|c||c|c|c|c|c|}
\n \\hline \n \\diaghead{NIgaaaa}{$s$}{$g$} ",
      for g in gs:
#         print "\t",g,
          print " & ", "\t",g,
      print "\\\\ \\hline \\hline",
      if (NB<NI):
          maxs=NB
      else:
          maxs=NI
      for s in range(1,maxs+1):
          print "\n %2d" % s,
          for g in gs:
            Ps = do_Qs(NB,NI,g,s)/do_q1C(NB,NI,g)
#           if (do_Qs(NB,NI,g,s) > q1C): print "ERROR: Qs and q1C are:",
"%8.6e"%do_Qs(NB,NI,g,s) , "%8.6e"%do_q1C(NB,NI,g)
#           print "%8.2e"%Qs ,
            print " & ", "%8.2e"%Ps ,
          print "\\\\ \\hline",
      print "\n \\end{tabular} \n \\end{table}"

parser=optparse.OptionParser()
help="""Usage:
%prog [options].
"""
parser.set_usage(help)
parser.add_option(
    "--NI",
    help="Valency of multivalent inhibitor",
    dest="NI",
    default=5
    )
parser.add_option(
```

```
    "--NB",
    help="Number of avaible binding sites on one capsid for one bound
multivalent inhibitor (e.g. [4,6])",
    dest="NBs",
    default=[4,6]
    )
parser.add_option(
    "--g",
    help="Binding free energy of only monovalnet inhibitor to one binding site",
    dest="gs",
    default=[-10,-1,-0.1,-0.01,-0.001]
    )

(options,arguments)=parser.parse_args()
do_probab(options.NI,options.NBs,options.gs)
```
